# Supplementary material for: Is dual use of nicotine products and cigarettes associated with smoking reduction and cessation behaviours? A prospective study in England
Source: BMJ Open. 2020 Mar 15;10(3):e036055. doi: 10.1136/bmjopen-2019-036055 (PMC7073794; doi:10.1136/bmjopen-2019-036055)
Supplement: Supplementary data [file bmjopen-2019-036055supp001.pdf]

Table S1. Comparison of respondents lost to follow-up and analytic sample

	**Baseline sample (*n*=2,318)**	**Lost to follow-up (*n*=1,905)**	**Study sample (*n*=413)**	***p****
**Sociodemographic characteristics**				
Age (years), % (*n*)				
16-31	31.0 (715)	33.9 (643)	17.4 (72)	<0.001
32-47	28.8 (665)	29.1 (552)	27.4 (113)	-
48-63	28.0 (646)	25.6 (485)	39.0 (161)	-
≥64	12.2 (281)	11.3 (214)	16.2 (67)	-
Female, % (*n*)	48.2 (1117)	48.3 (921)	47.5 (196)	0.743
Social grade C2DE, % (*n*)	58.9 (1366)	59.9 (1142)	54.2 (224)	0.032
**Smoking characteristics**				
Cigarettes per day, mean (SD)	11.61 (8.26)	11.29 (8.07)	13.06 (8.94)	<0.001
Strength of urges to smoke (range 0-5), mean (SD)	2.16 (1.08)	2.14 (1.09)	2.22 (1.03)	0.158
Non-daily smoker, % (*n*)	10.7 (248)	10.9 (207)	9.9 (41)	0.576
High motivation to quit, % (*n*)	27.6 (639)	27.8 (529)	26.6 (110)	0.627
Attempted to quit in past year, % (*n*)	50.9 (1159)	51.1 (955)	50.0 (204)	0.681
Product used, % (*n*)				
E-cigarettes	71.0 (1646)	70.8 (1348)	72.2 (298)	0.571
OTC NRT	29.0 (672)	29.2 (557)	27.8 (115)	-

Note. *comparison of respondents lost to follow-up and analytic sample, using t-test for means and chi-square for percentages. Age was categorised by standard deviation bands (16 years), with ≥80 collapsed into the 64-79 group due to low numbers (*n*=18 in baseline sample). SD = standard deviation; OTC NRT = over-the-counter nicotine replacement therapy.
