# Supplementary material for: Is dual use of nicotine products and cigarettes associated with smoking reduction and cessation behaviours? A prospective study in England
Source: BMJ Open. 2020 Mar 15;10(3):e036055. doi: 10.1136/bmjopen-2019-036055 (PMC7073794; doi:10.1136/bmjopen-2019-036055)
Supplement: Author's manuscript [file bmjopen-2019-036055.draft_revisions.pdf]

BMJ Open is committed to open peer review. As part of this commitment we make the peer review history of every article we publish publicly available.

When an article is published we post the peer reviewers' comments and the authors' responses online. We also post the versions of the paper that were used during peer review. These are the versions that the peer review comments apply to.

The versions of the paper that follow are the versions that were submitted during the peer review process. They are not the versions of record or the final published versions. They should not be cited or distributed as the published version of this manuscript.

BMJ Open is an open access journal and the full, final, typeset and author-corrected version of record of the manuscript is available on our site with no access controls, subscription charges or pay-per-view fees (http://bmjopen.bmj.com).

If you have any questions on BMJ Open's open peer review process please email info.bmjopen@bmj.com

BMJ Open

Prospective associations of dual use of nicotine products and cigarettes with smoking reduction and cessation behaviours in England

Journal:	*BMJ Open*
Manuscript ID	bmjopen-2019-036055
Article Type:	Original research
Date Submitted by the Author:	30-Nov-2019

Complete List of Authors:	Jackson, Sarah ; University College London, Epidemiology and Public Health;<br>Farrow, Emma; University College London<br>Brown, Jamie; University College London, Psychology & Language Sciences<br>Shahab, Lion; UCL, Dept of Epidemiology and Public Health

Keywords:	PUBLIC HEALTH, EPIDEMIOLOGY, RESPIRATORY MEDICINE (see Thoracic Medicine)

SCHOLARONE™
Manuscripts

I, the Submitting Author has the right to grant and does grant on behalf of all authors of the Work (as defined in the below author licence), an exclusive licence and/or a non-exclusive licence for contributions from authors who are: i) UK Crown employees; ii) where BMJ has agreed a CC-BY licence shall apply, and/or iii) in accordance with the terms applicable for US Federal Government officers or employees acting as part of their official duties; on a worldwide, perpetual, irrevocable, royalty-free basis to BMJ Publishing Group Ltd ("BMJ") its licensees and where the relevant Journal is co-owned by BMJ to the co-owners of the Journal, to publish the Work in this journal and any other BMJ products and to exploit all rights, as set out in our licence.

The Submitting Author accepts and understands that any supply made under these terms is made by BMJ to the Submitting Author unless you are acting as an employee on behalf of your employer or a postgraduate student of an affiliated institution which is paying any applicable article publishing charge ("APC") for Open Access articles. Where the Submitting Author wishes to make the Work available on an Open Access basis (and intends to pay the relevant APC), the terms of reuse of such Open Access shall be governed by a Creative Commons licence – details of these licences and which Creative Commons licence will apply to this Work are set out in our licence referred to above.

Other than as permitted in any relevant BMJ Author's Self Archiving Policies, I confirm this Work has not been accepted for publication elsewhere, is not being considered for publication elsewhere and does not duplicate material already published. I confirm all authors consent to publication of this Work and authorise the granting of this licence.

For peer review only

Prospective associations of dual use of nicotine products and cigarettes with smoking reduction and cessation behaviours in England

Sarah E. Jackson[1], Emma Farrow[1], Jamie Brown[1], Lion Shahab[1]

[1] Department of Behavioural Science and Health, University College London, London, UK

Corresponding author: Dr Sarah Jackson, Department of Behavioural Science and Health, University College London, 1-19 Torrington Place, London WC1E 7HB, UK. s.e.jackson@ucl.ac.uk +44 (0)207 679 8312

Word count: 3,648

For peer review only

Abstract

Objectives: To investigate associations of dual use of e-cigarettes and cigarettes with subsequent quitting activity (smoking reduction, quit attempts, and use of evidence-based cessation aids). To overcome potential confounding by factors associated with use of pharmacological support, we selected dual use of over-the-counter nicotine replacement therapy (OTC NRT) and cigarettes as a behavioural control.

Design: Prospective cohort study with 6-month follow-up.

Setting: England, 2014-2016.

Participants: 413 current smokers participating in the Smoking Toolkit Study, a representative survey of adults in England, who reported current use of e-cigarettes or OTC NRT and provided data at 6-month follow-up.

Main outcome measures: The exposure was dual use of e-cigarettes or OTC NRT at baseline. Outcomes were change in cigarette consumption, quit attempts, and use of evidence-based cessation aids during quit attempts over 6-month follow-up. Relevant sociodemographic and smoking characteristics were included as covariates.

Results: After adjustment for covariates, dual e-cigarette users smoked 2 fewer cigarettes per day at follow-up than at baseline compared with dual OTC NRT users (*B*=2.01, 95%CI -3.62; -0.39, *p*=0.015). While dual e-cigarette users had 18% lower odds than dual OTC NRT users to make a quit attempt at follow-up (RR 0.82, 95%CI 0.67-1.00, *p*=0.049), the groups did not differ in use of cessation aids (RR 1.06, 95%CI 0.93-1.21, *p*=0.388).

Conclusions: Dual use of e-cigarettes is associated with a greater reduction in cigarette consumption than dual use of OTC NRT. It may discourage a small proportion of users from making a quit attempt compared with dual OTC NRT use but it does not appear to undermine cessation aid use.

Key words: e-cigarettes; nicotine replacement therapy; dual use; smoking cessation; cigarette consumption

For peer review only - http://bmjopen.bmj.com/site/about/guidelines.xhtml

Strengths and limitations

- The use of a prospective study design allowed us to examine changes over time.
- However, the follow-up rate was low, and significant differences were noted between responders and non-responders on several variables.
- The reliance on self-reported data may increase measurement error or bias in key variables, such as quit attempts at 6-month follow-up.
- While analyses were adjusted for several potential confounders, there is the possibility of residual confounding by unmeasured variables.

For peer review only

For peer review only - http://bmjopen.bmj.com/site/about/guidelines.xhtml

Introduction

Cigarette smoking is a leading cause of morbidity and premature mortality, responsible for 96,000 deaths each year in the United Kingdom (UK) [1]. While substantial progress has been made in reducing smoking prevalence over recent years, around 15% of the UK adult population continue to smoke [2]. E-cigarettes are popular among smokers both as a smoking cessation aid and as a method of harm reduction among smokers not trying to quit [3,4]. As such, dual use of e-cigarettes and cigarettes ("dual e-cigarette use") is prevalent, with around 20% of the English smoker population also using e-cigarettes [5]. A growing body of evidence shows that e-cigarettes are substantially less harmful than cigarettes [6–9] and effective in promoting cessation [10–14]. However, concerns have been raised regarding unintended population-level consequences, including perpetuating nicotine addiction and reducing motivation to quit smoking [15,16], which may undermine the positive impact of e-cigarettes on population health [17].

Large-scale observational studies have examined associations between dual e-cigarette use and quit attempts in a real-world setting. These have largely [10,18,19], but not exclusively [11], observed a positive association, with e-cigarette use associated with a higher rate of quit attempts compared with non-use. There is also some evidence that e-cigarettes may help smokers to reduce the amount that they smoke. Cigarette consumption is a useful proxy for nicotine addiction, given nicotine addiction drives cigarette consumption [20] and smokers who are more addicted typically smoke a greater number of cigarettes per day [21]. In a Cochrane review of available evidence on e-cigarettes and smoking reduction, all included prospective cohort studies documented substantial reductions in cigarette consumption among e-cigarette users relative to non-users [22]. Experimental studies have shown similar effects even in smokers not intending to quit [23,24].

These findings suggest that a potential negative impact on quitting activity from the dual use of e-cigarettes and quitting activity appears unlikely. However, limitations of the existing literature include retrospective assessment of e-cigarette use [e.g., 18] and lack of adjustment for key confounders, such as motivation to quit [25,26]. Moreover, the comparison groups of most previous studies consisted of smokers not using e-cigarettes. This is not a suitable real-world control due to risk of confounding, for example by the comparatively higher levels of nicotine dependence and motivation to quit among smokers who use cessation aids [27].

For peer review only

For peer review only - http://bmjopen.bmj.com/site/about/guidelines.xhtml

Alternative licensed non-combustible nicotine products, such as over-the-counter nicotine replacement therapy (OTC NRT), provide a more appropriate comparator. Until the recent surge in popularity of e-cigarettes, NRT was the most commonly used quitting aid in England [28]. Like e-cigarettes, NRT is widely available to purchase without prescription and provides nicotine to help reduce cravings and withdrawal symptoms in periods of abstinence. Previous real-world comparisons of e-cigarette and OTC NRT have focused on use for, and outcomes of, smoking cessation [29], and supported the effectiveness of e-cigarettes. Further, a randomised controlled trial of e-cigarettes compared with OTC NRT and placebo e-cigarettes in smokers motivated to quit found that e-cigarette users were more likely to achieve reduced cigarette consumption and biochemically verified cessation than OTC NRT or placebo e-cigarette users [30]. Outside of a quit attempt, dual use of OTC NRT and cigarettes ("dual OTC NRT use") has been found to increase the real-world likelihood of quit attempts but not smoking reduction [31]. Understanding how dual e-cigarette use compares with dual OTC NRT use in its effects on smoking reduction and quit attempts in the real world, outside of clinical trials, is important for contextualising concerns about the risks of e-cigarette use.

Additionally, whether dual e-cigarette use is associated with cessation aid use in future quit attempts has significant implications for e-cigarettes' long-term impact. The use of cessation aids appears to promote quit success [14,32]. Thus, if the likelihood of cessation aid use in future quit attempts is increased, dual use of e-cigarettes would impact positively on population cessation rates. Currently, little is known of the association between use of aids on one occasion and their future use, although studies have found that use of medication (varenicline, bupropion, NRT) during a previous quit attempt is associated with a preference for the use of medication during a future quit attempt [33] and the use of the same cessation aid across quit attempts [34]. As such, use of e-cigarettes might be associated with greater likelihood of using cessation aids to support a future quit attempt.

The present study therefore aimed to examine prospective associations of dual e-cigarette use versus dual OTC NRT use, with subsequent smoking reduction, quit attempts, and use of evidence-based cessation aids. Specifically, we addressed the following research questions:

1. Is dual e-cigarette use at baseline associated with a) smoking reduction and b) quit attempts at 6-month follow-up compared with dual OTC NRT use?
2. Among those attempting to quit at the 6-month follow-up, is dual e-cigarette use at baseline associated with the use of evidence-based cessation aids compared with dual OTC NRT use?

For peer review only - http://bmjopen.bmj.com/site/about/guidelines.xhtml

Materials and Methods

Design

A prospective cohort study with data obtained from the Smoking Toolkit Study was conducted. The Smoking Toolkit Study is an ongoing national study of smoking prevalence and patterns in England. The Smoking Toolkit Study protocol has been described fully elsewhere [35], but briefly, it involves monthly face-to-face computer-assisted interviews with representative samples of adults in England. Households are selected using a hybrid of random location and simple quota sampling. From an initial approximately 170,000 grouped output areas, each compromising of approximately 300 households and stratified by ACORN characteristics (http://www.caci.co.uk/acorn/), grouped output areas are randomly selected and trained interviewers conduct interviews until quotas are reached. Approximately 1,700 adults (≥16 years) are interviewed at baseline per month (one per selected household), with samples shown to be representative of the population in England with regard to smoking prevalence and sociodemographic characteristics [35]. In certain waves (due to availability of funding), respondents consenting to re-contact at baseline have been followed up by telephone survey 6 months later. The University College London Ethics Committee provided ethical approval for the Smoking Toolkit Study (ID 0498/001) and all participants provided fully informed consent.

Sample

For the present study, we analysed data collected during baseline survey waves 90 (March 2014) through 120 (September 2016), which included a 6-month follow-up survey. We restricted our analytic sample to those participants who (i) reported smoking cigarettes (manufactured or hand-rolled) daily or occasionally at baseline, (ii) reported using either e-cigarettes or OTC NRT at baseline, and (iii) completed the 6-month follow-up survey.

Public and patient involvement

No patients or members of the public were involved in setting the research questions or the outcome measures, nor were they involved in the design and implementation of this specific study. There are no plans to involve patients in dissemination.

Measures

For peer review only - http://bmjopen.bmj.com/site/about/guidelines.xhtml

Explanatory variable

The explanatory variable was dual use of e-cigarette or OTC NRT (nicotine gum, nicotine patch, nicotine inhaler, nicotine lozenges/tablets). This was measured at baseline with three questions:

1. Which, if any, of the following are you currently using to help you cut down the amount you smoke?
2. Do you regularly use any of these in situations when you are not allowed to smoke?
3. Can I check, are you using any of the following at all for any reason?

The response options for each of these questions were: nicotine gum; nicotine lozenge; nicotine patch; nicotine inhaler/inhalator; another nicotine product; electronic cigarette; nicotine mouthspray; and other.

A binary variable was created, combining responses to these questions to distinguish between smokers who reported current use of e-cigarette and those who reported current use of NRT. Those using neither (73.5% of available sample) or both products (2.8%) were excluded.

Outcome variables

Our outcomes of interest were: (i) smoking reduction (change in *number of* cigarettes *smoked* per day) between baseline and 6-month follow-up, (ii) quit attempts between baseline and 6-month follow-up, (iii) use of cessation aids during the most recent quit attempt among those who tried to quit between baseline and 6-month follow-up.

Cigarette consumption was assessed by self-reports of the number of cigarettes the participant usually smoked per day or per week, according to their preference. Cigarettes per day was calculated as the number of cigarettes smoked per day or the number of cigarettes smoked per week divided by seven. Change in cigarettes per day was analysed as a continuous variable computed as 6-month follow-up cigarettes per day minus baseline cigarettes per day. Participants who reported being a non-smoker at follow-up were excluded from analyses of smoking reduction (*n*=77).

Quit attempts between baseline and 6-month follow-up were assessed with the question: "*How many serious attempts to stop smoking have you made in the last 6 months? By serious I mean you decided that you would try to make sure you never smoked again*." Quit attempts were coded 1 for those who reported at least one quit attempt and 0 for those who reported no quit attempts between baseline and follow-up.

For peer review only - http://bmjopen.bmj.com/site/about/guidelines.xhtml

Among those who made a quit attempt, use of evidence-based cessation aids (e-cigarettes, OTC NRT, face-to-face behavioural support, and prescribed medication [NRT, bupropion, or varenicline]) during the most recent attempt was assessed with the question: "*Which, if any, did you try to help you stop smoking during the most recent serious quit attempt?".* Response categories were collapsed to a binary variable indicating whether the quit attempt was aided or unaided.

Covariates

Potential confounding variables adjusted for in the analyses were selected a priori and measured at baseline.

Sociodemographic variables included: age, sex, and social grade. Age was analysed as a categorical variable banded by standard deviations, to account for the non-linear association between smoking and age. Social grade is an occupational index of socioeconomic position closely linked with smoking behaviour [36], categorised as ABC1, which includes managerial, administrative and professional and occupations, vs. C2DE, which includes semi-routine and routine occupations, manual occupations, never workers and long-term unemployed [37]).

An index of nicotine dependence measured at baseline was included as an additional adjustment in the cigarettes per day change analysis due to the positive correlation between levels of dependence and cigarettes per day [21]. This was measured with the item: "*How much of the time have you felt the urge to smoke in the past 24 hours?*". The response options ranged on a continuous scale from 0 ("*not at all")* to 5 ("*extremely strong"*).

Motivation to quit smoking measured at baseline was included as additional adjustment in the quit attempt analysis as it has been shown to be highly predictive of quit attempts [25,26]. This was measured using the Motivation to Stop Scale [38], a single item with 7 response categories coded as: 1) *"I don't want to stop smoking"; 2)* "*I think I should stop smoking but don't really want to*"; 3) "*I want to stop smoking but haven't thought about when*"; 4) "*I REALLY want to stop smoking but I don't know when I will*"; 5) "*I want to stop smoking and hope to soon*"; 6) "*I REALLY want to stop smoking and intend to in the next 3 months*"; 7) "*I REALLY want to stop smoking and intend to in the next month*". A binary variable was derived to indicate high motivation to quit (yes [score of 6 or 7] vs. no [score of 1-5]).

For peer review only - http://bmjopen.bmj.com/site/about/guidelines.xhtml

Having made a past-year quit attempt was also included as an additional adjustment in the quit attempt analysis as this has also been shown to be predictive of prospective quit attempts [39]. Participants were asked at baseline: "*How many serious attempts to stop smoking have you made in the last 12 months? By serious attempt I mean you decided that you would try to make sure you never smoked again. Please include any attempt that you are currently making, and please include any successful attempt within the last 12 months*." Quit attempts were coded 1 for those who reported at least one quit attempt and 0 for those who reported no quit attempts in the last 12 months.

Statistical analyses

The analysis plan was pre-registered on the Open Science Framework (https://osf.io/w6cxd/) and all analyses conducted in IBM SPSS Statistics v.24.

To assess the representativeness of the analytic sample, we compared responders and non-responders to the 6-month follow-up survey on baseline smoking and sociodemographic characteristics using independent *t*-tests for continuous variables, and chi-square tests for categorical variables. Within the analysed sample, we also compared dual e-cigarette users with dual OTC NRT users on the same characteristics.

We used linear regression to analyse the association between dual e-cigarette use versus dual OTC NRT use at baseline and (i) change in cigarettes per day, and log-binomial regression to analyse associations between e-cigarette use at baseline and (ii) quit attempts, and (iii) use of cessation aids; with and without adjustment for the above-mentioned covariates. In all models, dual OTC NRT use at baseline was the reference category.

As an unplanned sensitivity analysis, we replicated the adjusted quit attempt analysis substituting the full 7-level motivation to quit variable for the binary high/low motivation variable used in the primary analyses, to evaluate the robustness of this variable as an adjustment.

Results

Of 9,798 current smokers surveyed at baseline between March 2014 to September 2016, 2,318 (23.7%) reported dual e-cigarette use or dual OTC NRT use. Of the 2,318 smokers eligible at baseline, 413 (17.8%) completed the 6-month follow-up survey. Responders were significantly older and more socioeconomically advantaged than non-responders, and they reported a higher average cigarettes per day (Table S1). There

For peer review only - http://bmjopen.bmj.com/site/about/guidelines.xhtml

were no significant differences by sex, level of addiction, non-daily smoking, motivation to quit, past-year quit attempts, or product used (e-cigarettes vs. OTC NRT; Table S1).

Baseline characteristics of the analytic sample are shown in Table 1. Dual e-cigarette users were on average significantly younger than dual NRT users, were slightly heavier smokers, and were less likely to report high motivation to quit. The groups did not differ significantly on any other variable.

Smoking reduction

Between baseline and 6-month follow-up, mean cigarette consumption reduced by 0.06 (SD 6.57) cigarettes per day in dual e-cigarette users and increased by 2.09 (5.88) cigarettes per day in dual OTC NRT users. This difference remained significant after adjustment for age, sex, social grade, and level of nicotine addiction at baseline (Table 2).

Quit attempts

Between baseline and 6-month follow-up, 45.7% (*n*=186) made at least one serious attempt to quit smoking. The rate of quit attempts was significantly lower among dual e-cigarette users (41.8%, *n*=123) than dual OTC NRT users (55.8%, *n*=63) even after adjustment for age, sex, social grade, motivation to stop smoking, and past-year quit attempts at baseline (Table 2). A sensitivity analysis treating motivation to quit as a 7-level variable produced similar results, but the difference was no longer statistically significant (RR 0.82, 95% CI 0.66; 1.02, *p*=0.079).

Use of cessation aids

Of those who made at least one serious attempt to quit over the follow-up period (*n*=186), the majority (80.6%, *n*=150) reported having used an evidence-based cessation aid during their most recent quit attempt at 6-month follow-up. Use of cessation aids did not differ significantly between those using e-cigarette (82.9%, *n*=102) and those using OTC NRT at baseline (76.2%, *n*=48; Table 2), although there were some differences in the type of cessation aids used.

Smokers who reported dual e-cigarette use at baseline were most likely to subsequently use e-cigarettes as a cessation aid (74.7%), followed by prescription medication (11.7%; prescription NRT 5.2%, bupropion 0.6%, varenicline 5.8%), face-to-face behavioural support (5.8%) and OTC NRT (5.8%). By contrast, smokers who reported dual OTC NRT use at baseline were most likely to subsequently use prescription medication as

For peer review only - http://bmjopen.bmj.com/site/about/guidelines.xhtml

For peer review only

a cessation aid (39.5%; prescription NRT 30.3%, bupropion 2.6%, varenicline 6.6%), followed by OTC NRT (30.3%), face-to-face behavioural support (25.0%) and e-cigarettes (22.4%).

Discussion

In this study, we assessed prospective associations of dual use of e-cigarettes and cigarettes with smoking reduction, quit attempts, and use of evidence-based cessation aids compared with dual use of an alternative licensed nicotine product, OTC NRT. Dual e-cigarette users smoked on average 2 fewer cigarettes per day at 6-month follow-up than at baseline compared with dual OTC NRT users. However, dual e-cigarette use was associated with 18% reduced odds of making a serious quit attempt at 6-month follow-up relative to dual OTC NRT use, although there was no significant difference in the use of evidence-based cessation aids by those who attempted to quit.

The association we observed between e-cigarette use and smoking reduction is in line with previous research that has documented a greater reduction in cigarette consumption associated with use of e-cigarettes compared with use of OTC NRT by smokers intending to quit [30], and with use of e-cigarettes compared with non-use among smokers not intending to quit [24]. Even after adjustment for confounding variables, and exclusion of those who were non-smokers at follow-up (potentially causing the effect size to be underestimated), dual e-cigarette users' mean cigarettes per day was significantly lower at 6-month follow-up than at baseline compared with dual OTC NRT users'. This finding does not support concerns that e-cigarettes perpetuate smoking.

While benefits of dual e-cigarette use on cigarette consumption lend support to e-cigarettes as a harm reduction strategy, it is possible that smokers who may have otherwise initiated a quit attempt settled for smoking reduction instead. Consistent with this, our results show that dual e-cigarette users had 18% lower odds than dual OTC NRT users to make a serious quit attempt over the 6-month follow-up period. This finding is in agreement with evidence that smokers using e-cigarettes without the intention to quit are less likely to attempt to quit [40], but contrasts with previous real-world research that has found e-cigarettes to be more effective than OTC NRT in promoting cessation when used to support a quit attempt [29]. Importantly, the comparison in the present study was between dual e-cigarette use and dual OTC NRT use, rather than dual e-cigarette use and exclusive smoking. In a recent study that compared dual e-cigarette use, dual NRT use, and exclusive smoking, a hierarchical association with quit attempts was observed: dual e-cigarette use

For peer review only - http://bmjopen.bmj.com/site/about/guidelines.xhtml

was associated with a higher rate of quit attempts than exclusive smoking but a lower rate than NRT [41]. Taken together, the evidence appears to support concerns that e-cigarettes may divert cessation attempts [42], but only relative to NRT.

A possible explanation is that, compared with NRT, e-cigarettes may reduce motivation to quit, for example, by allowing temporary abstinence in situations where smoking is prohibited [43]. Previous research has indicated that while many smokers report that e-cigarettes assist in resisting the urge to smoke in such situations [44], OTC NRT is less effective [45,46]. In support of the importance of motivation in the association between dual e-cigarette use and quit attempts, while our primary analyses showed a significantly lower rate of quit attempts among e-cigarette users even after adjustment a dichotomous (high/low) indicator of motivation to quit, this result was not significant in a sensitivity analysis using a more nuanced 7-level measure of motivation. Further research into the mediators of the relationship between e-cigarette use and quit attempts is required to shed light on the reasons for this potential adverse consequence of e-cigarette use and inform targeted interventions to prevent dual e-cigarette use from deterring smokers from quitting.

Despite dual e-cigarette users being significantly less likely than dual OTC NRT users to make a quit attempt, those who attempted to quit did not differ significantly in their use of evidence-based cessation aids. This suggests that dual e-cigarette use does not undermine their use of evidence-based support. With use of cessation aids a strong predictor of quit success [14], this is important for maximising the chance that a quit attempt is successful. The finding that the majority (~80%) of those dual using e-cigarettes or OTC NRT subsequently made a quit attempt using evidence based-cessation aids is consistent with evidence that previous use of a cessation aid increases preference and likelihood of their future use [33,34]. To maximise statistical power in a limited sample, evidence-based cessation aids (e-cigarettes, NRT, face-to-face behavioural support, and prescription medication) were combined for analysis, but descriptive data indicated that dual e-cigarette users tended to subsequently opt for e-cigarettes as a cessation aid, while dual OTC NRT users tended to use prescription medication or OTC NRT. Consistent with the fact that stop smoking services in England typically provide a combination of prescription medication and behavioural support, use of face-to-face behavioural support was substantially higher in the group who reported dual OTC NRT use at baseline.

A key strength of the study is the prospective design. However, there were also several limitations. Smoking reduction and quitting behaviour were examined only over a 6-month period, and thus replication of results with longer-term studies is required. The follow-up rate was also low, with just 17.8% of eligible baseline

For peer review only - http://bmjopen.bmj.com/site/about/guidelines.xhtml

participants completing the 6-month follow-up survey, and significant differences were noted between responders and non-responders on several variables. The reliance on self-reported data may increase measurement error or bias in key variables, such as quit attempts at 6-month follow-up. For example, research has found that recall of failed quit attempts is low [47], which may reduce the observed effectiveness of harm reduction aids for quit attempts. If recall bias differs between dual users of e-cigarettes and OTC NRT, this may create spurious associations. Further, associations with use of support during quit attempts may be biased if unaided quit attempts were recalled less frequently than aided [48]. Finally, while analyses were adjusted for several potential confounders, there is the possibility of residual confounding by unmeasured variables, such as the frequency [49] and duration [50] of e-cigarette/NRT use and the type of e-cigarette/NRT used. Future research with more detailed assessment of e-cigarette use could provide useful insights.

Conclusions

These findings lend some support to concerns that e-cigarettes may discourage quitting activity relative to other popular nicotine products, showing slightly lower odds of quit attempts among dual users of e-cigarettes and cigarettes compared with dual users of OTC NRT and cigarettes. However, they provide no evidence of an adverse effect of e-cigarette use on cigarette consumption; on the contrary, dual e-cigarette use was associated with greater smoking reduction compared with dual OTC NRT use. In addition, dual use of e-cigarettes did not appear to undermine use of evidence-based cessation aids during quit attempts.

For peer review only

Contributorship statement

EF, JB and LS conceived and designed the study. SJ and EF analysed the data and drafted the manuscript. JB and LS provided critical revisions. All authors read and approved the submitted version.

Competing interests

LS has received a research grant and honoraria for a talk and travel expenses from manufacturers of smoking cessation medications (Pfizer and Johnson & Johnson). JB has received unrestricted research funding from Pfizer, who manufacture smoking cessation medications. All authors declare no financial links with tobacco companies or e-cigarette manufacturers or their representatives. All other authors declare no competing interests.

Funding

Cancer Research UK funded data collection (C1417/A22962; C44576/A19501) and SJ & JB's salary (C1417/A22962). SJ's salary was also supported by the Economic and Social Research Council (ES/R005990/1). The funders had no final role in the study design; in the collection, analysis and interpretation of data; in the writing of the report; or in the decision to submit the paper for publication. All researchers listed as authors are independent from the funders and all final decisions about the research were taken by the investigators and were unrestricted.

Data sharing statement

Data are available upon request.

For peer review only - http://bmjopen.bmj.com/site/about/guidelines.xhtml

For peer review only

References

1. Action on Smoking and Health. *Facts at a Glance - Key Smoking Statistics*.; 2018. file:///N:/Downloads/Facts-at-a-Glance-12-Sept-2018-FINAL.pdf. Accessed June 26, 2019.

2. Office for National Statistics. *Adult Smoking Habits in the UK: 2017*.; 2018. https://www.ons.gov.uk/peoplepopulationandcommunity/healthandsocialcare/healthandlifeexpectancies/bulletins/adultsmokinghabitsingreatbritain/2017#the-proportion-who-are-current-smokers-in-the-uk-its-constituent-countries-and-local-areas-2011-to-2017. Accessed August 22, 2018.

3. Beard E, Brown J, Michie S, West R. Is prevalence of e-cigarette and nicotine replacement therapy use among smokers associated with average cigarette consumption in England? A time-series analysis. *BMJ Open*. 2018;8(6):e016046. doi:10.1136/bmjopen-2017-016046

4. Patel D, Davis KC, Cox S, et al. Reasons for current E-cigarette use among U.S. adults. *Prev Med*. 2016;93:14-20. doi:10.1016/j.ypmed.2016.09.011

5. West R, Proudfoot H, Beard E, Brown J. *Electronic Cigarettes in England - Latest Trends*. Smoking in England; 2019. http://www.smokinginengland.info/latest-statistics/. Accessed May 26, 2019.

6. McNeill A, Brose LS, Calder R, Bauld L, Robson D. Evidence review of e-cigarettes and heated tobacco products 2018. *Rep Comm Public Health Engl Lond Public Health Engl*. 2018;6.

7. Goniewicz ML, Gawron M, Smith DM, Peng M, Jacob P, Benowitz NL. Exposure to Nicotine and Selected Toxicants in Cigarette Smokers Who Switched to Electronic Cigarettes: A Longitudinal Within-Subjects Observational Study. *Nicotine Tob Res*. 2017;19(2):160-167. doi:10.1093/ntr/ntw160

8. McRobbie H, Phillips A, Goniewicz ML, et al. Effects of Switching to Electronic Cigarettes with and without Concurrent Smoking on Exposure to Nicotine, Carbon Monoxide, and Acrolein. *Cancer Prev Res Phila Pa*. 2015;8(9):873-878. doi:10.1158/1940-6207.CAPR-15-0058

9. Shahab L, Goniewicz ML, Blount BC, et al. Nicotine, Carcinogen, and Toxin Exposure in Long-Term E-Cigarette and Nicotine Replacement Therapy Users: A Cross-sectional Study. *Ann Intern Med*. 2017;166(6):390-400. doi:10.7326/M16-1107

10. Zhu S-H, Zhuang Y-L, Wong S, Cummins SE, Tedeschi GJ. E-cigarette use and associated changes in population smoking cessation: evidence from US current population surveys. *BMJ*. 2017;358:j3262. doi:10.1136/bmj.j3262

11. Beard E, West R, Michie S, Brown J. Association between electronic cigarette use and changes in quit attempts, success of quit attempts, use of smoking cessation pharmacotherapy, and use of stop smoking services in England: time series analysis of population trends. *BMJ*. 2016;354:i4645. doi:10.1136/bmj.i4645

12. Hartmann-Boyce J, Begh R, Aveyard P. Electronic cigarettes for smoking cessation. *BMJ*. 2018;360:j5543. doi:10.1136/bmj.j5543

For peer review only - http://bmjopen.bmj.com/site/about/guidelines.xhtml

13. Hajek P, Phillips-Waller A, Przulj D, et al. A Randomized Trial of E-Cigarettes versus Nicotine-Replacement Therapy. *N Engl J Med*. 2019;380(7):629-637. doi:10.1056/NEJMoa1808779

14. Jackson SE, Kotz D, West R, Brown J. Moderators of real-world effectiveness of smoking cessation aids: a population study. *Addiction*. 2019.

15. Glantz SA, Bareham DW. E-Cigarettes: Use, Effects on Smoking, Risks, and Policy Implications. *Annu Rev Public Health*. 2018;39:215-235. doi:10.1146/annurev-publhealth-040617-013757

16. Bartter T. Electronic Cigarettes: Aggregate Harm. *Ann Intern Med*. 2015;163(1):59-60. doi:10.7326/M15-0450

17. Levy DT, Borland R, Villanti AC, et al. The Application of a Decision-Theoretic Model to Estimate the Public Health Impact of Vaporized Nicotine Product Initiation in the United States. *Nicotine Tob Res Off J Soc Res Nicotine Tob*. 2017;19(2):149-159. doi:10.1093/ntr/ntw158

18. Gmel G, Baggio S, Mohler-Kuo M, Daeppen J-B, Studer J. E-cigarette use in young Swiss men: is vaping an effective way of reducing or quitting smoking? *Swiss Med Wkly*. 2016;146:w14271. doi:10.4414/smw.2016.14271

19. Brose LS, Hitchman SC, Brown J, West R, McNeill A. Is the use of electronic cigarettes while smoking associated with smoking cessation attempts, cessation and reduced cigarette consumption? A survey with a 1-year follow-up. *Addiction*. 2015;110(7):1160-1168. doi:10.1111/add.12917

20. Le Houezec J, McNeill A, Britton J. Tobacco, nicotine and harm reduction. *Drug Alcohol Rev*. 2011;30(2):119-123. doi:10.1111/j.1465-3362.2010.00264.x

21. Donny EC, Griffin KM, Shiffman S, Sayette MA. The relationship between cigarette use, nicotine dependence, and craving in laboratory volunteers. *Nicotine Tob Res Off J Soc Res Nicotine Tob*. 2008;10(5):934-942. doi:10.1080/14622200802133681

22. McRobbie H, Bullen C, Hartmann-Boyce J, Hajek P. Electronic cigarettes for smoking cessation and reduction. *Cochrane Database Syst Rev*. 2014;(12):CD010216. doi:10.1002/14651858.CD010216.pub2

23. Polosa R, Caponnetto P, Morjaria JB, Papale G, Campagna D, Russo C. Effect of an electronic nicotine delivery device (e-Cigarette) on smoking reduction and cessation: a prospective 6-month pilot study. *BMC Public Health*. 2011;11:786. doi:10.1186/1471-2458-11-786

24. Adriaens K, Van Gucht D, Declerck P, Baeyens F. Effectiveness of the electronic cigarette: An eight-week Flemish study with six-month follow-up on smoking reduction, craving and experienced benefits and complaints. *Int J Environ Res Public Health*. 2014;11(11):11220-11248. doi:10.3390/ijerph111111220

25. Borland R, Yong H-H, Balmford J, et al. Motivational factors predict quit attempts but not maintenance of smoking cessation: findings from the International Tobacco Control Four country project. *Nicotine Tob Res Off J Soc Res Nicotine Tob*. 2010;12 Suppl:S4-11. doi:10.1093/ntr/ntq050

For peer review only

For peer review only - http://bmjopen.bmj.com/site/about/guidelines.xhtml

26. Zhou X, Nonnemaker J, Sherrill B, Gilsenan AW, Coste F, West R. Attempts to quit smoking and relapse: Factors associated with success or failure from the ATTEMPT cohort study. *Addict Behav*. 2009;34(4):365-373. doi:10.1016/j.addbeh.2008.11.013

27. Shi Y, Pierce JP, White M, et al. E-cigarette use and smoking reduction or cessation in the 2010/2011 TUS-CPS longitudinal cohort. *BMC Public Health*. 2016;16(1):1105. doi:10.1186/s12889-016-3770-x

28. West R, Brown J, Shahab L. Written evidence submitted by University College London, Tobacco and Alcohol Research Group (UTARG) (ECG0047). http://data.parliament.uk/writtenevidence/committeeevidence.svc/evidencedocument/science-and-technology-committee/ecigarettes/written/75276.html. Published 2017. Accessed October 11, 2018.

29. Brown J, Beard E, Kotz D, Michie S, West R. Real-world effectiveness of e-cigarettes when used to aid smoking cessation: a cross-sectional population study. *Addiction*. 2014;109(9):1531-1540. doi:10.1111/add.12623

30. Bullen C, Howe C, Laugesen M, et al. Electronic cigarettes for smoking cessation: a randomised controlled trial. *The Lancet*. 2013;382(9905):1629-1637. doi:10.1016/S0140-6736(13)61842-5

31. Beard E, McNeill A, Aveyard P, Fidler J, Michie S, West R. Association between use of nicotine replacement therapy for harm reduction and smoking cessation: a prospective study of English smokers. *Tob Control*. 2013;22(2):118-122. doi:10.1136/tobaccocontrol-2011-050007

32. Kotz D, Brown J, West R. 'Real-world'effectiveness of smoking cessation treatments: a population study. *Addiction*. 2014;109(3):491–499.

33. Thomas D, Abramson MJ, Bonevski B, et al. Quitting experiences and preferences for a future quit attempt: a study among inpatient smokers. *BMJ Open*. 2015;5(4):e006959. doi:10.1136/bmjopen-2014-006959

34. Ferguson SG, Brown J, Frandsen M, West R. Associations between use of pharmacological aids in a smoking cessation attempt and subsequent quitting activity: a population study. *Addict Abingdon Engl*. 2015;110(3):513-518. doi:10.1111/add.12795

35. Fidler JA, Shahab L, West O, et al. "The smoking toolkit study": a national study of smoking and smoking cessation in England. *BMC Public Health*. 2011;11:479. doi:10.1186/1471-2458-11-479

36. Kotz D, West R. Explaining the social gradient in smoking cessation: it's not in the trying, but in the succeeding. *Tob Control*. 2009;18(1):43-46. doi:10.1136/tc.2008.025981

37. National Readership Survey. Social grade - definitions and discriminatory power. 2007. http://www.nrs.co.uk/lifestyle.html. Accessed October 1, 2012.

38. Kotz D, Brown J, West R. Predictive validity of the Motivation To Stop Scale (MTSS): A single-item measure of motivation to stop smoking. *Drug Alcohol Depend*. 2013;128(1):15-19. doi:10.1016/j.drugalcdep.2012.07.012

For peer review only - http://bmjopen.bmj.com/site/about/guidelines.xhtml

39. Hyland A, Laux FL, Higbee C, et al. Cigarette purchase patterns in four countries and the relationship with cessation: findings from the International Tobacco Control (ITC) Four Country Survey. *Tob Control*. 2006;15 Suppl 3:iii59-64. doi:10.1136/tc.2005.012203

40. Al-Delaimy WK, Myers MG, Leas EC, Strong DR, Hofstetter CR. E-cigarette use in the past and quitting behavior in the future: a population-based study. *Am J Public Health*. 2015;105(6):1213-1219. doi:10.2105/AJPH.2014.302482

41. Jackson SE, Shahab L, West R, Brown J. Associations between dual use of e-cigarettes and smoking quit attempts, success, and quit rates: a prospective study of smokers in England. *Addict Behav*. in press.

42. Selya AS, Rose JS, Dierker L, Hedeker D, Mermelstein RJ. Evaluating the mutual pathways among electronic cigarette use, conventional smoking and nicotine dependence. *Addict Abingdon Engl*. 2018;113(2):325-333. doi:10.1111/add.14013

43. Villanti AC, Feirman SP, Niaura RS, et al. How do we determine the impact of e-cigarettes on cigarette smoking cessation or reduction? Review and recommendations for answering the research question with scientific rigor. *Addiction*. 2018;113(3):391-404. doi:10.1111/add.14020

44. Etter J-F. Electronic cigarettes: a survey of users. *BMC Public Health*. 2010;10(1):231. doi:10.1186/1471-2458-10-231

45. Etter JF, Perneger TV. Attitudes toward nicotine replacement therapy in smokers and ex-smokers in the general public. *Clin Pharmacol Ther*. 2001;69(3):175-183. doi:10.1067/mcp.2001.113722

46. Brown J, Hajek P, McRobbie H, et al. Cigarette craving and withdrawal symptoms during temporary abstinence and the effect of nicotine gum. *Psychopharmacology (Berl)*. 2013;229(1):209-218. doi:10.1007/s00213-013-3100-2

47. Berg CJ, An LC, Kirch M, et al. Failure to report attempts to quit smoking. *Addict Behav*. 2010;35(10):900-904. doi:10.1016/j.addbeh.2010.06.009

48. Borland R, Partos TR, Cummings KM. Systematic Biases in Cross-sectional Community Studies may Underestimate the Effectiveness of Stop-Smoking Medications. *Nicotine Tob Res*. 2012;14(12):1483-1487. doi:10.1093/ntr/nts002

49. Levy DT, Yuan Z, Luo Y, Abrams DB. The Relationship of E-Cigarette Use to Cigarette Quit Attempts and Cessation: Insights From a Large, Nationally Representative U.S. Survey. *Nicotine Tob Res*. 2018;20(8):931-939. doi:10.1093/ntr/ntx166

50. Zhuang Y-L, Cummins SE, Sun JY, Zhu S-H. Long-term e-cigarette use and smoking cessation: a longitudinal study with US population. *Tob Control*. 2016;25(Suppl 1):i90-i95. doi:10.1136/tobaccocontrol-2016-053096

For peer review only

For peer review only - http://bmjopen.bmj.com/site/about/guidelines.xhtml

Tables

Table 1. Baseline sample characteristics

	Dual users of e-cigarettes (*n*=298)	Dual users of OTC NRT (*n*=115)	*p*
**Sociodemographic characteristics**			
Age (years), % (*n*)			
16-31	20.1 (60)	10.4 (12)	0.006
32-47	29.9 (89)	20.9 (24)	-
48-63	35.9 (107)	47.0 (54)	-
≥64	14.1 (42)	21.7 (25)	-
Female, % (*n*)	46.0 (137)	51.2 (59)	0.331
Social grade C2DE, % (*n*)	52.7 (157)	58.3 (67)	0.308
**Smoking characteristics**			
Cigarettes per day, mean (SD)	13.52 (9.56)	11.82 (6.94)	0.049
Strength of urges to smoke (range 0-5), mean (SD)	2.26 (1.03)	2.13 (1.05)	0.266
Non-daily smoker, % (*n*)	9.7 (29)	10.4 (12)	0.830
High motivation to quit, % (*n*)	23.2 (69)	35.8 (41)	0.010
Attempted to quit in past year, % (*n*)	48.6 (144)	53.6 (60)	0.375

Note. Age was categorised by standard deviation bands (16 years), with ≥80 collapsed into the 64-79 group due to low numbers. SD = standard deviation; OTC NRT = over-the-counter nicotine replacement therapy.

For peer review only

For peer review only - http://bmjopen.bmj.com/site/about/guidelines.xhtml

Table 2. Associations between use of e-cigarettes compared with use of over-the-counter nicotine replacement therapy and (i) smoking reduction, (ii) quit attempts, and (iii) use of cessation aids over six-month follow-up

	**Unadjusted**			**Adjusted***		
	**B (SE)**	**95% CI**	***p***	**B (SE)**	**95% CI**	***p***
Change in cigarettes per day	-2.15 (0.81)	-3.74; -0.56	0.008	-2.01 (0.82)	-3.62; -0.39	0.015
	**RR**	**95% CI**	***p***	**RR**	**95% CI**	***p***
Quit attempts	0.75	0.61; 0.93	0.008	0.82	0.67; 1.00	0.049
Use of evidence-based cessation aids	1.04	0.92; 1.19	0.524	1.06	0.93; 1.21	0.388

Note. *All models adjusted for age, sex and social grade. The model for change in cigarettes per day also adjusted for strength of urges to smoke; the model for quit attempts also adjusted for motivation to stop smoking and past-year quit attempts. SE = standard error, CI = confidence interval, RR = risk ratio.

For peer review only

Supporting information captions

Table S1. Comparison of respondents lost to follow-up and analytic sample

For peer review only

For peer review only - http://bmjopen.bmj.com/site/about/guidelines.xhtml

Table S1. Comparison of respondents lost to follow-up and analytic sample

	Baseline sample (*n*=2,318)	Lost to follow-up (*n*=1,905)	Study sample (*n*=413)	*p**
**Sociodemographic characteristics**				
Age (years), % (*n*)				
16-31	31.0 (715)	33.9 (643)	17.4 (72)	<0.001
32-47	28.8 (665)	29.1 (552)	27.4 (113)	-
48-63	28.0 (646)	25.6 (485)	39.0 (161)	-
≥64	12.2 (281)	11.3 (214)	16.2 (67)	-
Female, % (*n*)	48.2 (1117)	48.3 (921)	47.5 (196)	0.743
Social grade C2DE, % (*n*)	58.9 (1366)	59.9 (1142)	54.2 (224)	0.032
**Smoking characteristics**				
Cigarettes per day, mean (SD)	11.61 (8.26)	11.29 (8.07)	13.06 (8.94)	<0.001
Strength of urges to smoke (range 0-5), mean (SD)	2.16 (1.08)	2.14 (1.09)	2.22 (1.03)	0.158
Non-daily smoker, % (*n*)	10.7 (248)	10.9 (207)	9.9 (41)	0.576
High motivation to quit, % (*n*)	27.6 (639)	27.8 (529)	26.6 (110)	0.627
Attempted to quit in past year, % (*n*)	50.9 (1159)	51.1 (955)	50.0 (204)	0.681
Product used, % (*n*)				
E-cigarettes	71.0 (1646)	70.8 (1348)	72.2 (298)	0.571
OTC NRT	29.0 (672)	29.2 (557)	27.8 (115)	-

Note. *comparison of respondents lost to follow-up and analytic sample, using t-test for means and chi-square for percentages. Age was categorised by standard deviation bands (16 years), with ≥80 collapsed into the 64-79 group due to low numbers (*n*=18 in baseline sample). SD = standard deviation; OTC NRT = over-the-counter nicotine replacement therapy.

For peer review only

STROBE Statement—Checklist of items that should be included in reports of ***cross-sectional studies***

	Item No	Recommendation	Page
**Title and abstract**	1	(*a*) Indicate the study's design with a commonly used term in the title or the abstract	1
		(*b*) Provide in the abstract an informative and balanced summary of what was done and what was found	2
**Introduction**			
Background/rationale	2	Explain the scientific background and rationale for the investigation being reported	3
Objectives	3	State specific objectives, including any prespecified hypotheses	4
**Methods**			
Study design	4	Present key elements of study design early in the paper	5
Setting	5	Describe the setting, locations, and relevant dates, including periods of recruitment, exposure, follow-up, and data collection	5
Participants	6	(*a*) Give the eligibility criteria, and the sources and methods of selection of participants	5
Variables	7	Clearly define all outcomes, exposures, predictors, potential confounders, and effect modifiers. Give diagnostic criteria, if applicable	6
Data sources/ measurement	8*	For each variable of interest, give sources of data and details of methods of assessment (measurement). Describe comparability of assessment methods if there is more than one group	6
Bias	9	Describe any efforts to address potential sources of bias	7
Study size	10	Explain how the study size was arrived at	8
Quantitative variables	11	Explain how quantitative variables were handled in the analyses. If applicable, describe which groupings were chosen and why	6
Statistical methods	12	(*a*) Describe all statistical methods, including those used to control for confounding	8
		(*b*) Describe any methods used to examine subgroups and interactions	8
		(*c*) Explain how missing data were addressed	8
		(*d*) If applicable, describe analytical methods taking account of sampling strategy	na
		(*e*) Describe any sensitivity analyses	8
**Results**			
Participants	13*	(a) Report numbers of individuals at each stage of study—eg numbers potentially eligible, examined for eligibility, confirmed eligible, included in the study, completing follow-up, and analysed	8
		(b) Give reasons for non-participation at each stage	8
		(c) Consider use of a flow diagram	na
Descriptive data	14*	(a) Give characteristics of study participants (eg demographic, clinical, social) and information on exposures and potential confounders	9
		(b) Indicate number of participants with missing data for each variable of interest	na
Outcome data	15*	Report numbers of outcome events or summary measures	Tables
Main results	16	(*a*) Give unadjusted estimates and, if applicable, confounder-adjusted estimates and their precision (eg, 95% confidence interval). Make clear which confounders were adjusted for and why they were included	Tables

For peer review only - http://bmjopen.bmj.com/site/about/guidelines.xhtml

		(*b*) Report category boundaries when continuous variables were categorized	na
		(*c*) If relevant, consider translating estimates of relative risk into absolute risk for a meaningful time period	na
Other analyses	17	Report other analyses done—eg analyses of subgroups and interactions, and sensitivity analyses	9
**Discussion**			
Key results	18	Summarise key results with reference to study objectives	10
Limitations	19	Discuss limitations of the study, taking into account sources of potential bias or imprecision. Discuss both direction and magnitude of any potential bias	12
Interpretation	20	Give a cautious overall interpretation of results considering objectives, limitations, multiplicity of analyses, results from similar studies, and other relevant evidence	12
Generalisability	21	Discuss the generalisability (external validity) of the study results	12
**Other information**			
Funding	22	Give the source of funding and the role of the funders for the present study and, if applicable, for the original study on which the present article is based	13

*Give information separately for exposed and unexposed groups.

Note: An Explanation and Elaboration article discusses each checklist item and gives methodological background and published examples of transparent reporting. The STROBE checklist is best used in conjunction with this article (freely available on the Web sites of PLoS Medicine at http://www.plosmedicine.org/, Annals of Internal Medicine at http://www.annals.org/, and Epidemiology at http://www.epidem.com/). Information on the STROBE Initiative is available at www.strobe-statement.org.

For peer review only

For peer review only - http://bmjopen.bmj.com/site/about/guidelines.xhtml

BMJ Open

Is dual use of nicotine products and cigarettes associated with smoking reduction and cessation behaviours? A prospective study in England

Journal:	*BMJ Open*
Manuscript ID	bmjopen-2019-036055.R1
Article Type:	Original research
Date Submitted by the Author:	28-Jan-2020
Complete List of Authors:	Jackson, Sarah ; University College London, Epidemiology and Public Health;<br>Farrow, Emma; University College London<br>Brown, Jamie; University College London, Psychology & Language Sciences<br>Shahab, Lion; UCL, Dept of Epidemiology and Public Health
**Primary Subject Heading**:	Public health
Secondary Subject Heading:	Epidemiology
Keywords:	PUBLIC HEALTH, EPIDEMIOLOGY, RESPIRATORY MEDICINE (see Thoracic Medicine)

SCHOLARONE™
Manuscripts

BMJ

I, the Submitting Author has the right to grant and does grant on behalf of all authors of the Work (as defined in the below author licence), an exclusive licence and/or a non-exclusive licence for contributions from authors who are: i) UK Crown employees; ii) where BMJ has agreed a CC-BY licence shall apply, and/or iii) in accordance with the terms applicable for US Federal Government officers or employees acting as part of their official duties; on a worldwide, perpetual, irrevocable, royalty-free basis to BMJ Publishing Group Ltd ("BMJ") its licensees and where the relevant Journal is co-owned by BMJ to the co-owners of the Journal, to publish the Work in this journal and any other BMJ products and to exploit all rights, as set out in our licence.

The Submitting Author accepts and understands that any supply made under these terms is made by BMJ to the Submitting Author unless you are acting as an employee on behalf of your employer or a postgraduate student of an affiliated institution which is paying any applicable article publishing charge ("APC") for Open Access articles. Where the Submitting Author wishes to make the Work available on an Open Access basis (and intends to pay the relevant APC), the terms of reuse of such Open Access shall be governed by a Creative Commons licence – details of these licences and which Creative Commons licence will apply to this Work are set out in our licence referred to above.

Other than as permitted in any relevant BMJ Author's Self Archiving Policies, I confirm this Work has not been accepted for publication elsewhere, is not being considered for publication elsewhere and does not duplicate material already published. I confirm all authors consent to publication of this Work and authorise the granting of this licence.

For peer review only

Is dual use of nicotine products and cigarettes associated with smoking reduction and cessation behaviours? A prospective study in England

Sarah E. Jackson[1], Emma Farrow[1], Jamie Brown[1], Lion Shahab[1]

[1] Department of Behavioural Science and Health, University College London, London, UK

Corresponding author: Dr Sarah Jackson, Department of Behavioural Science and Health, University College London, 1-19 Torrington Place, London WC1E 7HB, UK. s.e.jackson@ucl.ac.uk +44 (0)207 679 8312

Word count: 3,648

For peer review only - http://bmjopen.bmj.com/site/about/guidelines.xhtml

Abstract

Objectives: To investigate associations of dual use of e-cigarettes and cigarettes with subsequent quitting activity (smoking reduction, quit attempts, and use of evidence-based cessation aids). To overcome potential confounding by factors associated with use of pharmacological support, we selected dual use of over-the-counter nicotine replacement therapy (OTC NRT) and cigarettes as a behavioural control.

Design: Prospective cohort study with 6-month follow-up.

Setting: England, 2014-2016.

Participants: 413 current smokers participating in the Smoking Toolkit Study, a representative survey of adults in England, who reported current use of e-cigarettes or OTC NRT and provided data at 6-month follow-up.

Main outcome measures: The exposure was dual use of e-cigarettes or OTC NRT at baseline. Outcomes were change in cigarette consumption, quit attempts, and use of evidence-based cessation aids during quit attempts over 6-month follow-up. Relevant sociodemographic and smoking characteristics were included as covariates.

Results: After adjustment for covariates, dual e-cigarette users smoked 2 fewer cigarettes per day at follow-up than at baseline compared with dual OTC NRT users (B=2.01, 95%CI -3.62; -0.39, p=0.015). While dual e-cigarette users had 18% lower odds than dual OTC NRT users to make a quit attempt at follow-up (RR 0.82, 95%CI 0.67-1.00, p=0.049), the groups did not differ in use of cessation aids (RR 1.06, 95%CI 0.93-1.21, p=0.388).

Conclusions: Dual use of e-cigarettes is associated with a greater reduction in cigarette consumption than dual use of OTC NRT. It may discourage a small proportion of users from making a quit attempt compared with dual OTC NRT use but it does not appear to undermine use of evidence-based cessation aids.

Key words: e-cigarettes; nicotine replacement therapy; dual use; smoking cessation; cigarette consumption

For peer review only

For peer review only - http://bmjopen.bmj.com/site/about/guidelines.xhtml

Strengths and limitations

- The use of a prospective study design allowed us to examine changes over time.
- However, the follow-up rate was low, and significant differences were noted between responders and non-responders on several variables.
- The reliance on self-reported data may increase measurement error or bias in key variables, such as quit attempts at 6-month follow-up.
- While analyses were adjusted for several potential confounders, there is the possibility of residual confounding by unmeasured variables.

For peer review only

For peer review only - http://bmjopen.bmj.com/site/about/guidelines.xhtml

Introduction

Cigarette smoking is a leading cause of morbidity and premature mortality, responsible for 96,000 deaths each year in the United Kingdom (UK) [1]. While substantial progress has been made in reducing smoking prevalence over recent years, around 15% of the UK adult population continue to smoke [2]. E-cigarettes are popular among smokers both as a smoking cessation aid and as a method of harm reduction among smokers not trying to quit [3,4]. As such, dual use of e-cigarettes and cigarettes ("dual e-cigarette use") is prevalent, with around 20% of the English smoker population also using e-cigarettes [5]. A growing body of evidence shows that e-cigarettes are substantially less harmful than cigarettes [6–9] and effective in promoting cessation [10–14]. However, concerns have been raised regarding unintended population-level consequences, including perpetuating nicotine addiction and reducing motivation to quit smoking [15,16], which may undermine the positive impact of e-cigarettes on population health [17].

Large-scale observational studies have examined associations between dual e-cigarette use and quit attempts in a real-world setting. These have largely [10,18,19], but not exclusively [11], observed a positive association, with e-cigarette use associated with a higher rate of quit attempts compared with non-use. There is also some evidence that e-cigarettes may help smokers to reduce the amount that they smoke. Cigarette consumption is a useful proxy for nicotine addiction, given nicotine addiction drives cigarette consumption [20] and smokers who are more addicted typically smoke a greater number of cigarettes per day [21]. In a Cochrane review of available evidence on e-cigarettes and smoking reduction, all included prospective cohort studies documented substantial reductions in cigarette consumption among e-cigarette users relative to non-users [22]. Experimental studies have shown similar effects even in smokers not intending to quit [23,24]. This is notable given that many dual e-cigarette users do not use e-cigarettes for cessation [25,26].

These findings suggest that a potential negative impact on quitting activity from the dual use of e-cigarettes and quitting activity appears unlikely. However, limitations of the existing literature include retrospective assessment of e-cigarette use [e.g., 18] and lack of adjustment for key confounders, such as motivation to quit [27,28]. Moreover, the comparison groups of most previous studies consisted of smokers not using e-cigarettes. This is not a suitable real-world control due to risk of confounding, for example by the comparatively higher levels of nicotine dependence and motivation to quit among smokers who use cessation aids [29].

For peer review only - http://bmjopen.bmj.com/site/about/guidelines.xhtml

Alternative licensed non-combustible nicotine products, such as over-the-counter nicotine replacement therapy (OTC NRT), provide a more appropriate comparator. Until the recent surge in popularity of e-cigarettes, NRT was the most commonly used quitting aid in England [30]. Like e-cigarettes, NRT is widely available to purchase without prescription and provides nicotine to help reduce cravings and withdrawal symptoms in periods of abstinence. Previous real-world comparisons of e-cigarette and OTC NRT have focused on use for, and outcomes of, smoking cessation [31], and supported the effectiveness of e-cigarettes. Further, a randomised controlled trial of e-cigarettes compared with OTC NRT and placebo e-cigarettes in smokers motivated to quit found that e-cigarette users were more likely to achieve reduced cigarette consumption and biochemically verified cessation than OTC NRT or placebo e-cigarette users [32]. Outside of a quit attempt, dual use of OTC NRT and cigarettes (“dual OTC NRT use”) has been found to increase the real-world likelihood of quit attempts but not smoking reduction [33]. Understanding how dual e-cigarette use compares with dual OTC NRT use in its effects on smoking reduction and quit attempts in the real world, outside of clinical trials, is important for contextualising concerns about the risks of e-cigarette use.

Additionally, whether dual e-cigarette use is associated with cessation aid use in future quit attempts has significant implications for e-cigarettes’ long-term impact. The use of cessation aids appears to promote quit success [14,34]. Thus, if the likelihood of cessation aid use in future quit attempts is increased, dual use of e-cigarettes would impact positively on population cessation rates. Currently, little is known of the association between use of aids on one occasion and their future use, although studies have found that use of medication (varenicline, bupropion, NRT) during a previous quit attempt is associated with a preference for the use of medication during a future quit attempt [35] and the use of the same cessation aid across quit attempts [36]. As such, use of e-cigarettes might be associated with greater likelihood of using cessation aids to support a future quit attempt.

The present study therefore aimed to examine prospective associations of dual e-cigarette use versus dual OTC NRT use, with subsequent smoking reduction, quit attempts, and use of evidence-based cessation aids. Specifically, we addressed the following research questions:

1. Is dual e-cigarette use at baseline associated with a) smoking reduction and b) quit attempts at 6-month follow-up compared with dual OTC NRT use?
2. Among those attempting to quit at the 6-month follow-up, is dual e-cigarette use at baseline associated with the use of evidence-based cessation aids compared with dual OTC NRT use?

For peer review only - http://bmjopen.bmj.com/site/about/guidelines.xhtml

Materials and Methods

Design

A prospective cohort study with data obtained from the Smoking Toolkit Study was conducted. The Smoking Toolkit Study is an ongoing national study of smoking prevalence and patterns in England. The Smoking Toolkit Study protocol has been described fully elsewhere [37], but briefly, it involves monthly face-to-face computer-assisted interviews with representative samples of adults in England. Households are selected using a hybrid of random location and simple quota sampling. From an initial approximately 170,000 grouped output areas, each compromising of approximately 300 households and stratified by ACORN characteristics (http://www.caci.co.uk/acorn/), grouped output areas are randomly selected and trained interviewers conduct interviews until quotas are reached. Approximately 1,700 adults (≥16 years) are interviewed at baseline per month (one per selected household), with samples shown to be representative of the population in England with regard to smoking prevalence and sociodemographic characteristics [37]. In certain waves (due to availability of funding), respondents consenting to re-contact at baseline have been followed up by telephone survey 6 months later. The University College London Ethics Committee provided ethical approval for the Smoking Toolkit Study (ID 0498/001) and all participants provided fully informed consent.

Sample

For the present study, we analysed data collected during baseline survey waves 90 (March 2014) through 120 (September 2016), which included a 6-month follow-up survey. We restricted our analytic sample to those participants who (i) reported smoking cigarettes (manufactured or hand-rolled) daily or occasionally at baseline, (ii) reported using either e-cigarettes or OTC NRT at baseline, and (iii) completed the 6-month follow-up survey.

Public and patient involvement

No patients or members of the public were involved in setting the research questions or the outcome measures, nor were they involved in the design and implementation of this specific study. There are no plans to involve patients in dissemination.

Measures

For peer review only - http://bmjopen.bmj.com/site/about/guidelines.xhtml

Explanatory variable

The explanatory variable was dual use of e-cigarette or OTC NRT (nicotine gum, nicotine patch, nicotine inhaler, nicotine lozenges/tablets). This was measured at baseline with three questions:

1. Which, if any, of the following are you currently using to help you cut down the amount you smoke?
2. Do you regularly use any of these in situations when you are not allowed to smoke?
3. Can I check, are you using any of the following at all for any reason?

The response options for each of these questions were: nicotine gum; nicotine lozenge; nicotine patch; nicotine inhaler/inhalator; another nicotine product; electronic cigarette; nicotine mouthspray; and other.

A binary variable was created, combining responses to these questions to distinguish between smokers who reported current use of e-cigarette and those who reported current use of NRT. Those using neither (73.5% of available sample) or both products (2.8%) were excluded.

Outcome variables

Our outcomes of interest were: (i) smoking reduction (change in *number of* cigarettes *smoked* per day) between baseline and 6-month follow-up, (ii) quit attempts between baseline and 6-month follow-up, (iii) use of cessation aids during the most recent quit attempt among those who tried to quit between baseline and 6-month follow-up.

Cigarette consumption was assessed by self-reports of the number of cigarettes the participant usually smoked per day or per week, according to their preference. Cigarettes per day was calculated as the number of cigarettes smoked per day or the number of cigarettes smoked per week divided by seven. Change in cigarettes per day was analysed as a continuous variable computed as 6-month follow-up cigarettes per day minus baseline cigarettes per day. Participants who reported being a non-smoker at follow-up were excluded from analyses of smoking reduction (*n*=77).

Quit attempts between baseline and 6-month follow-up were assessed with the question: "*How many serious attempts to stop smoking have you made in the last 6 months? By serious I mean you decided that you would try to make sure you never smoked again*." Quit attempts were coded 1 for those who reported at least one quit attempt and 0 for those who reported no quit attempts between baseline and follow-up.

For peer review only - http://bmjopen.bmj.com/site/about/guidelines.xhtml

Among those who made a quit attempt, use of evidence-based cessation aids (e-cigarettes, OTC NRT, face-to-face behavioural support, and prescribed medication [NRT, bupropion, or varenicline]) during the most recent attempt was assessed with the question: "*Which, if any, did you try to help you stop smoking during the most recent serious quit attempt?".* Response categories were collapsed to a binary variable indicating whether the quit attempt was aided or unaided.

Covariates

Potential confounding variables adjusted for in the analyses were selected a priori and measured at baseline.

Sociodemographic variables included: age, sex, and social grade. Age was analysed as a categorical variable banded by standard deviations, to account for the non-linear association between smoking and age. Social grade is an occupational index of socioeconomic position closely linked with smoking behaviour [38], categorised as ABC1, which includes managerial, administrative and professional and occupations, vs. C2DE, which includes semi-routine and routine occupations, manual occupations, never workers and long-term unemployed [39]).

An index of nicotine dependence measured at baseline was included as an additional adjustment in the cigarettes per day change analysis due to the positive correlation between levels of dependence and cigarettes per day [21]. This was measured with the item: "*How much of the time have you felt the urge to smoke in the past 24 hours?*". The response options ranged on a continuous scale from 0 ("*not at all")* to 5 ("*extremely strong*").

Motivation to quit smoking measured at baseline was included as additional adjustment in the quit attempt analysis as it has been shown to be highly predictive of quit attempts [27,28]. This was measured using the Motivation to Stop Scale [40], a single item with 7 response categories coded as: 1) *"I don't want to stop smoking"; 2)* "*I think I should stop smoking but don't really want to*"; 3) "*I want to stop smoking but haven't thought about when*"; 4) "*I REALLY want to stop smoking but I don't know when I will*"; 5) "*I want to stop smoking and hope to soon*"; 6) "*I REALLY want to stop smoking and intend to in the next 3 months*"; 7) "*I REALLY want to stop smoking and intend to in the next month*". A binary variable was derived to indicate high motivation to quit (yes [score of 6 or 7] vs. no [score of 1-5]).

For peer review only - http://bmjopen.bmj.com/site/about/guidelines.xhtml

Having made a past-year quit attempt was also included as an additional adjustment in the quit attempt analysis as this has also been shown to be predictive of prospective quit attempts [41]. Participants were asked at baseline: "*How many serious attempts to stop smoking have you made in the last 12 months? By serious attempt I mean you decided that you would try to make sure you never smoked again. Please include any attempt that you are currently making, and please include any successful attempt within the last 12 months*." Quit attempts were coded 1 for those who reported at least one quit attempt and 0 for those who reported no quit attempts in the last 12 months.

Statistical analyses

The analysis plan was pre-registered on the Open Science Framework (https://osf.io/w6cxd/) and all analyses conducted in IBM SPSS Statistics v.24.

To assess the representativeness of the analytic sample, we compared responders and non-responders to the 6-month follow-up survey on baseline smoking and sociodemographic characteristics using independent *t*-tests for continuous variables, and chi-square tests for categorical variables. Within the analysed sample, we also compared dual e-cigarette users with dual OTC NRT users on the same characteristics.

We used linear regression to analyse the association between dual e-cigarette use versus dual OTC NRT use at baseline and (i) change in cigarettes per day, and log-binomial regression to analyse associations between e-cigarette use at baseline and (ii) quit attempts, and (iii) use of cessation aids; with and without adjustment for the above-mentioned covariates. We also added an unplanned analysis following peer review which tested the association with use of behavioural support and/or prescription medication in the most recent quit attempt, in order to disentangle continuation of use of e-cigarettes or OTC NRT from a behavioural outcome that could be conceptualised as a more distinctive step toward cessation. In all models, dual OTC NRT use at baseline was the reference category.

As an unplanned sensitivity analysis, we replicated the adjusted quit attempt analysis substituting the full 7-level motivation to quit variable for the binary high/low motivation variable used in the primary analyses, to evaluate the robustness of this variable as an adjustment.

Results

For peer review only - http://bmjopen.bmj.com/site/about/guidelines.xhtml

Of 9,798 current smokers surveyed at baseline between March 2014 to September 2016, 2,318 (23.7%) reported dual e-cigarette use or dual OTC NRT use. Of the 2,318 smokers eligible at baseline, 413 (17.8%) completed the 6-month follow-up survey. Responders were significantly older and more socioeconomically advantaged than non-responders, and they reported a higher average cigarettes per day (Table S1). There were no significant differences by sex, level of addiction, non-daily smoking, motivation to quit, past-year quit attempts, or product used (e-cigarettes vs. OTC NRT; Table S1).

Baseline characteristics of the analytic sample are shown in Table 1. Dual e-cigarette users were on average significantly younger than dual NRT users, were slightly heavier smokers, and were less likely to report high motivation to quit. The groups did not differ significantly on any other variable.

Smoking reduction

Between baseline and 6-month follow-up, mean cigarette consumption reduced by 0.06 (SD 6.57) cigarettes per day in dual e-cigarette users and increased by 2.09 (5.88) cigarettes per day in dual OTC NRT users. This difference remained significant after adjustment for age, sex, social grade, and level of nicotine addiction at baseline (Table 2).

Quit attempts

Between baseline and 6-month follow-up, 45.7% (*n*=186) made at least one serious attempt to quit smoking. The rate of quit attempts was significantly lower among dual e-cigarette users (41.8%, *n*=123) than dual OTC NRT users (55.8%, *n*=63) even after adjustment for age, sex, social grade, motivation to stop smoking, and past-year quit attempts at baseline (Table 2). A sensitivity analysis treating motivation to quit as a 7-level variable produced similar results, but the difference was no longer statistically significant (RR 0.82, 95% CI 0.66; 1.02, *p*=0.079).

Use of cessation aids

Of those who made at least one serious attempt to quit over the follow-up period (*n*=186), the majority (80.6%, *n*=150) reported having used an evidence-based cessation aid during their most recent quit attempt at 6-month follow-up. Overall, use of cessation aids did not differ significantly between those using e-cigarette (82.9%, *n*=102) and those using OTC NRT at baseline (76.2%, *n*=48; Table 2), although there were some differences in the type of cessation aids used.

For peer review only - http://bmjopen.bmj.com/site/about/guidelines.xhtml

Smokers who reported dual e-cigarette use at baseline were most likely to subsequently use e-cigarettes as a cessation aid (74.7%), followed by prescription medication (11.7%; prescription NRT 5.2%, bupropion 0.6%, varenicline 5.8%), face-to-face behavioural support (5.8%) and OTC NRT (5.8%). By contrast, smokers who reported dual OTC NRT use at baseline were most likely to subsequently use prescription medication as a cessation aid (39.5%; prescription NRT 30.3%, bupropion 2.6%, varenicline 6.6%), followed by OTC NRT (30.3%), face-to-face behavioural support (25.0%) and e-cigarettes (22.4%). When analysed separately from e-cigarettes and OTC NRT, dual e-cigarette users who made a serious quit attempt were significantly less likely than dual OTC NRT users who made a quit attempt to report using behavioural support or prescription medication during their most recent quit attempt at 6-month follow-up (Table 2).

Discussion

In this study, we assessed prospective associations of dual use of e-cigarettes and cigarettes with smoking reduction, quit attempts, and use of evidence-based cessation aids compared with dual use of an alternative licensed nicotine product, OTC NRT. Dual e-cigarette users smoked on average 2 fewer cigarettes per day at 6-month follow-up than at baseline compared with dual OTC NRT users. However, dual e-cigarette use was associated with 18% reduced odds of making a serious quit attempt at 6-month follow-up relative to dual OTC NRT use. Although there was no significant difference in the use of evidence-based cessation aids by those who attempted to quit, dual e-cigarette use was associated with 73% reduced odds of using behavioural support or prescription medication specifically.

The association we observed between e-cigarette use and smoking reduction is in line with previous research that has documented a greater reduction in cigarette consumption associated with use of e-cigarettes compared with use of OTC NRT by smokers intending to quit [32], and with use of e-cigarettes compared with non-use among smokers not intending to quit [24]. In our sample, dual e-cigarette users reported smoking more cigarettes per day than dual OTC NRT users at baseline. Even after adjustment for confounding variables, and exclusion of those who were non-smokers at follow-up (potentially causing the effect size to be underestimated), dual e-cigarette users' mean cigarettes per day was significantly lower at 6-month follow-up than at baseline compared with dual OTC NRT users', leaving the two groups with similar consumption at follow-up. This finding does not support concerns that e-cigarettes perpetuate smoking, but rather may be associated with reduced smoking among heavier smokers.

For peer review only - http://bmjopen.bmj.com/site/about/guidelines.xhtml

While benefits of dual e-cigarette use on cigarette consumption lend support to e-cigarettes as a harm reduction strategy, it is possible that smokers who may have otherwise initiated a quit attempt settled for smoking reduction instead. Consistent with this, our results show that dual e-cigarette users had 18% lower odds than dual OTC NRT users to make a serious quit attempt over the 6-month follow-up period. This finding is in agreement with evidence that smokers using e-cigarettes without the intention to quit are less likely to attempt to quit [42], but contrasts with previous real-world research that has found e-cigarettes to be more effective than OTC NRT in promoting cessation when used to support a quit attempt [31]. Importantly, the comparison in the present study was between dual e-cigarette use and dual OTC NRT use, rather than dual e-cigarette use and exclusive smoking. In a recent study that compared dual e-cigarette use, dual NRT use, and exclusive smoking, a hierarchical association with quit attempts was observed: dual e-cigarette use was associated with a higher rate of quit attempts than exclusive smoking but a lower rate than NRT [43]. Taken together, the evidence appears to support concerns that e-cigarettes may divert cessation attempts [44], but only relative to NRT.

A possible explanation is that, compared with NRT, e-cigarettes may reduce motivation to quit, for example, by allowing temporary abstinence in situations where smoking is prohibited [45]. Previous research has indicated that while many smokers report that e-cigarettes assist in resisting the urge to smoke in such situations [46], OTC NRT is less effective [47,48]. Alternatively, the difference in the rate of quit attempts may simply reflect the fact that dual e-cigarette users were less likely than dual OTC NRT users to be motivated to quit to begin with. In support of the importance of motivation in the association between dual e-cigarette use and quit attempts, while our primary analyses showed a significantly lower rate of quit attempts among e-cigarette users even after adjustment for a dichotomous (high/low) indicator of motivation to quit, this result was not significant in a sensitivity analysis using a more nuanced 7-level measure of motivation. Further research into the mediators of the relationship between e-cigarette use and quit attempts is required to shed light on the reasons for this potential adverse consequence of e-cigarette use and inform targeted interventions to prevent dual e-cigarette use from deterring smokers from quitting.

For peer review only

Despite dual e-cigarette users being significantly less likely than dual OTC NRT users to make a quit attempt, those who attempted to quit did not differ significantly in their use of evidence-based cessation aids. This suggests that dual e-cigarette use does not undermine their use of evidence-based support. With use of cessation aids a strong predictor of quit success [14], this is important for maximising the chance that a quit attempt is successful. The finding that the majority (~80%) of those dual using e-cigarettes or OTC NRT subsequently made a quit attempt using evidence based-cessation aids is consistent with evidence that

For peer review only - http://bmjopen.bmj.com/site/about/guidelines.xhtml

previous use of a cessation aid increases preference and likelihood of their future use [35,36]. To maximise statistical power in a limited sample, evidence-based cessation aids (e-cigarettes, NRT, face-to-face behavioural support, and prescription medication) were combined for analysis, but descriptive data indicated that dual e-cigarette users tended to subsequently opt for e-cigarettes as a cessation aid, while dual OTC NRT users tended to use prescription medication or OTC NRT. Consistent with the fact that stop smoking services in England typically provide a combination of prescription medication and behavioural support, use of face-to-face behavioural support was substantially higher in the group who reported dual OTC NRT use at baseline. An unplanned analysis that focused on use of behavioural support or prescription medication revealed that dual e-cigarette users were substantially less likely than dual OTC NRT users to report use of these cessation aids. It is not clear from our results how far this reflects greater (perceived) effectiveness of e-cigarettes than OTC NRT or lower motivation to quit causing dual e-cigarette users being less likely to seek out alternative support. This is something that should be explored in future research.

A key strength of the study is the prospective design. However, there were also several limitations. Smoking reduction and quitting behaviour were examined only over a 6-month period, and thus replication of results with longer-term studies is required. The follow-up rate was also low, with just 17.8% of eligible baseline participants completing the 6-month follow-up survey, and significant differences were noted between responders and non-responders on several variables. Thus, the results may not be representative of all dual e-cigarette users in England and may not generalise to other samples. The reliance on self-reported data may increase measurement error or bias in key variables, such as quit attempts at 6-month follow-up. For example, research has found that recall of failed quit attempts is low [49], which may reduce the observed effectiveness of harm reduction aids for quit attempts. If recall bias differs between dual users of e-cigarettes and OTC NRT, this may create spurious associations. Further, associations with use of support during quit attempts may be biased if unaided quit attempts were recalled less frequently than aided [50]. Additionally, while analyses were adjusted for several potential confounders, there is the possibility of residual confounding by unmeasured variables, such as the frequency [51] and duration [52] of e-cigarette/NRT use and the type of e-cigarette/NRT used. Future research with more detailed assessment of e-cigarette use could provide useful insights. Finally, this study was conducted in England, where e-cigarettes are regulated under the European Union's Tobacco Products Directive, which includes minimum standards for the safety and quality of all e-cigarettes and refill containers and restricts e-liquids to a nicotine strength of no more than 20mg/ml. Our results may not generalise to other countries that do not have regulations for quality

For peer review only - http://bmjopen.bmj.com/site/about/guidelines.xhtml

control or restrictions on nicotine concentration; particularly if higher levels of nicotine in e-cigarettes influence their effectiveness as a cessation aid.

Conclusions

These findings lend some support to concerns that e-cigarettes may discourage quitting activity relative to other popular nicotine products, showing slightly lower odds of quit attempts among dual users of e-cigarettes and cigarettes compared with dual users of OTC NRT and cigarettes. However, they provide no evidence of an adverse effect of e-cigarette use on cigarette consumption; on the contrary, dual e-cigarette use was associated with greater smoking reduction compared with dual OTC NRT use. In addition, dual use of e-cigarettes did not appear to undermine use of evidence-based cessation aids during quit attempts but was associated with reduced odds of using behavioural support or prescription medication specifically.

For peer review only

Contributorship statement

EF, JB and LS conceived and designed the study. SJ and EF analysed the data and drafted the manuscript. JB and LS provided critical revisions. All authors read and approved the submitted version.

Competing interests

LS has received a research grant and honoraria for a talk and travel expenses from manufacturers of smoking cessation medications (Pfizer and Johnson & Johnson). JB has received unrestricted research funding from Pfizer, who manufacture smoking cessation medications. All authors declare no financial links with tobacco companies or e-cigarette manufacturers or their representatives. All other authors declare no competing interests.

Funding

Cancer Research UK funded data collection (C1417/A22962; C44576/A19501) and SJ & JB's salary (C1417/A22962). SJ's salary was also supported by the Economic and Social Research Council (ES/R005990/1). The funders had no final role in the study design; in the collection, analysis and interpretation of data; in the writing of the report; or in the decision to submit the paper for publication. All researchers listed as authors are independent from the funders and all final decisions about the research were taken by the investigators and were unrestricted.

Data sharing statement

Data are available upon request.

For peer review only - http://bmjopen.bmj.com/site/about/guidelines.xhtml

References

1. Action on Smoking and Health. *Facts at a Glance - Key Smoking Statistics*.; 2018. file:///N:/Downloads/Facts-at-a-Glance-12-Sept-2018-FINAL.pdf. Accessed June 26, 2019.

2. Office for National Statistics. *Adult Smoking Habits in the UK: 2017*.; 2018. https://www.ons.gov.uk/peoplepopulationandcommunity/healthandsocialcare/healthandlifeexpectancies/bulletins/adultsmokinghabitsingreatbritain/2017#the-proportion-who-are-current-smokers-in-the-uk-its-constituent-countries-and-local-areas-2011-to-2017. Accessed August 22, 2018.

3. Beard E, Brown J, Michie S, West R. Is prevalence of e-cigarette and nicotine replacement therapy use among smokers associated with average cigarette consumption in England? A time-series analysis. *BMJ Open*. 2018;8(6):e016046. doi:10.1136/bmjopen-2017-016046

4. Patel D, Davis KC, Cox S, et al. Reasons for current E-cigarette use among U.S. adults. *Prev Med*. 2016;93:14-20. doi:10.1016/j.ypmed.2016.09.011

5. West R, Proudfoot H, Beard E, Brown J. *Electronic Cigarettes in England - Latest Trends*. Smoking in England; 2019. http://www.smokinginengland.info/latest-statistics/. Accessed May 26, 2019.

6. McNeill A, Brose LS, Calder R, Bauld L, Robson D. Evidence review of e-cigarettes and heated tobacco products 2018. *Rep Comm Public Health Engl Lond Public Health Engl*. 2018;6.

7. Goniewicz ML, Gawron M, Smith DM, Peng M, Jacob P, Benowitz NL. Exposure to Nicotine and Selected Toxicants in Cigarette Smokers Who Switched to Electronic Cigarettes: A Longitudinal Within-Subjects Observational Study. *Nicotine Tob Res*. 2017;19(2):160-167. doi:10.1093/ntr/ntw160

8. McRobbie H, Phillips A, Goniewicz ML, et al. Effects of Switching to Electronic Cigarettes with and without Concurrent Smoking on Exposure to Nicotine, Carbon Monoxide, and Acrolein. *Cancer Prev Res Phila Pa*. 2015;8(9):873-878. doi:10.1158/1940-6207.CAPR-15-0058

9. Shahab L, Goniewicz ML, Blount BC, et al. Nicotine, Carcinogen, and Toxin Exposure in Long-Term E-Cigarette and Nicotine Replacement Therapy Users: A Cross-sectional Study. *Ann Intern Med*. 2017;166(6):390-400. doi:10.7326/M16-1107

10. Zhu S-H, Zhuang Y-L, Wong S, Cummins SE, Tedeschi GJ. E-cigarette use and associated changes in population smoking cessation: evidence from US current population surveys. *BMJ*. 2017;358:j3262. doi:10.1136/bmj.j3262

11. Beard E, West R, Michie S, Brown J. Association between electronic cigarette use and changes in quit attempts, success of quit attempts, use of smoking cessation pharmacotherapy, and use of stop smoking services in England: time series analysis of population trends. *BMJ*. 2016;354:i4645. doi:10.1136/bmj.i4645

12. Hartmann-Boyce J, Begh R, Aveyard P. Electronic cigarettes for smoking cessation. *BMJ*. 2018;360:j5543. doi:10.1136/bmj.j5543

For peer review only - http://bmjopen.bmj.com/site/about/guidelines.xhtml

13. Hajek P, Phillips-Waller A, Przulj D, et al. A Randomized Trial of E-Cigarettes versus Nicotine-Replacement Therapy. *N Engl J Med*. 2019;380(7):629-637. doi:10.1056/NEJMoa1808779

14. Jackson SE, Kotz D, West R, Brown J. Moderators of real-world effectiveness of smoking cessation aids: a population study. *Addiction*. 2019.

15. Glantz SA, Bareham DW. E-Cigarettes: Use, Effects on Smoking, Risks, and Policy Implications. *Annu Rev Public Health*. 2018;39:215-235. doi:10.1146/annurev-publhealth-040617-013757

16. Bartter T. Electronic Cigarettes: Aggregate Harm. *Ann Intern Med*. 2015;163(1):59-60. doi:10.7326/M15-0450

17. Levy DT, Borland R, Villanti AC, et al. The Application of a Decision-Theoretic Model to Estimate the Public Health Impact of Vaporized Nicotine Product Initiation in the United States. *Nicotine Tob Res Off J Soc Res Nicotine Tob*. 2017;19(2):149-159. doi:10.1093/ntr/ntw158

18. Gmel G, Baggio S, Mohler-Kuo M, Daeppen J-B, Studer J. E-cigarette use in young Swiss men: is vaping an effective way of reducing or quitting smoking? *Swiss Med Wkly*. 2016;146:w14271. doi:10.4414/smw.2016.14271

19. Brose LS, Hitchman SC, Brown J, West R, McNeill A. Is the use of electronic cigarettes while smoking associated with smoking cessation attempts, cessation and reduced cigarette consumption? A survey with a 1-year follow-up. *Addiction*. 2015;110(7):1160-1168. doi:10.1111/add.12917

20. Le Houezec J, McNeill A, Britton J. Tobacco, nicotine and harm reduction. *Drug Alcohol Rev*. 2011;30(2):119-123. doi:10.1111/j.1465-3362.2010.00264.x

21. Donny EC, Griffin KM, Shiffman S, Sayette MA. The relationship between cigarette use, nicotine dependence, and craving in laboratory volunteers. *Nicotine Tob Res Off J Soc Res Nicotine Tob*. 2008;10(5):934-942. doi:10.1080/14622200802133681

22. McRobbie H, Bullen C, Hartmann-Boyce J, Hajek P. Electronic cigarettes for smoking cessation and reduction. *Cochrane Database Syst Rev*. 2014;(12):CD010216. doi:10.1002/14651858.CD010216.pub2

23. Polosa R, Caponnetto P, Morjaria JB, Papale G, Campagna D, Russo C. Effect of an electronic nicotine delivery device (e-Cigarette) on smoking reduction and cessation: a prospective 6-month pilot study. *BMC Public Health*. 2011;11:786. doi:10.1186/1471-2458-11-786

24. Adriaens K, Van Gucht D, Declerck P, Baeyens F. Effectiveness of the electronic cigarette: An eight-week Flemish study with six-month follow-up on smoking reduction, craving and experienced benefits and complaints. *Int J Environ Res Public Health*. 2014;11(11):11220-11248. doi:10.3390/ijerph111111220

25. Mantey DS, Cooper MR, Loukas A, Perry CL. E-cigarette Use and Cigarette Smoking Cessation among Texas College Students. *Am J Health Behav*. 2017;41(6):750-759. doi:10.5993/AJHB.41.6.9

26. Romijnders KAGJ, van Osch L, de Vries H, Talhout R. Perceptions and Reasons Regarding E-Cigarette Use among Users and Non-Users: A Narrative Literature Review. *Int J Environ Res Public Health*. 2018;15(6). doi:10.3390/ijerph15061190

For peer review only

27. Borland R, Yong H-H, Balmford J, et al. Motivational factors predict quit attempts but not maintenance of smoking cessation: findings from the International Tobacco Control Four country project. *Nicotine Tob Res Off J Soc Res Nicotine Tob*. 2010;12 Suppl:S4-11. doi:10.1093/ntr/ntq050

28. Zhou X, Nonnemaker J, Sherrill B, Gilsenan AW, Coste F, West R. Attempts to quit smoking and relapse: Factors associated with success or failure from the ATTEMPT cohort study. *Addict Behav*. 2009;34(4):365-373. doi:10.1016/j.addbeh.2008.11.013

29. Shi Y, Pierce JP, White M, et al. E-cigarette use and smoking reduction or cessation in the 2010/2011 TUS-CPS longitudinal cohort. *BMC Public Health*. 2016;16(1):1105. doi:10.1186/s12889-016-3770-x

30. West R, Brown J, Shahab L. Written evidence submitted by University College London, Tobacco and Alcohol Research Group (UTARG) (ECG0047). http://data.parliament.uk/writtenevidence/committeeevidence.svc/evidencedocument/science-and-technology-committee/ecigarettes/written/75276.html. Published 2017. Accessed October 11, 2018.

31. Brown J, Beard E, Kotz D, Michie S, West R. Real-world effectiveness of e-cigarettes when used to aid smoking cessation: a cross-sectional population study. *Addiction*. 2014;109(9):1531-1540. doi:10.1111/add.12623

32. Bullen C, Howe C, Laugesen M, et al. Electronic cigarettes for smoking cessation: a randomised controlled trial. *The Lancet*. 2013;382(9905):1629-1637. doi:10.1016/S0140-6736(13)61842-5

33. Beard E, McNeill A, Aveyard P, Fidler J, Michie S, West R. Association between use of nicotine replacement therapy for harm reduction and smoking cessation: a prospective study of English smokers. *Tob Control*. 2013;22(2):118-122. doi:10.1136/tobaccocontrol-2011-050007

34. Kotz D, Brown J, West R. 'Real-world'effectiveness of smoking cessation treatments: a population study. *Addiction*. 2014;109(3):491–499.

35. Thomas D, Abramson MJ, Bonevski B, et al. Quitting experiences and preferences for a future quit attempt: a study among inpatient smokers. *BMJ Open*. 2015;5(4):e006959. doi:10.1136/bmjopen-2014-006959

36. Ferguson SG, Brown J, Frandsen M, West R. Associations between use of pharmacological aids in a smoking cessation attempt and subsequent quitting activity: a population study. *Addict Abingdon Engl*. 2015;110(3):513-518. doi:10.1111/add.12795

37. Fidler JA, Shahab L, West O, et al. "The smoking toolkit study": a national study of smoking and smoking cessation in England. *BMC Public Health*. 2011;11:479. doi:10.1186/1471-2458-11-479

38. Kotz D, West R. Explaining the social gradient in smoking cessation: it's not in the trying, but in the succeeding. *Tob Control*. 2009;18(1):43-46. doi:10.1136/tc.2008.025981

39. National Readership Survey. Social grade - definitions and discriminatory power. 2007. http://www.nrs.co.uk/lifestyle.html. Accessed October 1, 2012.

For peer review only

For peer review only - http://bmjopen.bmj.com/site/about/guidelines.xhtml

40. Kotz D, Brown J, West R. Predictive validity of the Motivation To Stop Scale (MTSS): A single-item measure of motivation to stop smoking. *Drug Alcohol Depend*. 2013;128(1):15-19. doi:10.1016/j.drugalcdep.2012.07.012

41. Hyland A, Laux FL, Higbee C, et al. Cigarette purchase patterns in four countries and the relationship with cessation: findings from the International Tobacco Control (ITC) Four Country Survey. *Tob Control*. 2006;15 Suppl 3:iii59-64. doi:10.1136/tc.2005.012203

42. Al-Delaimy WK, Myers MG, Leas EC, Strong DR, Hofstetter CR. E-cigarette use in the past and quitting behavior in the future: a population-based study. *Am J Public Health*. 2015;105(6):1213-1219. doi:10.2105/AJPH.2014.302482

43. Jackson SE, Shahab L, West R, Brown J. Associations between dual use of e-cigarettes and smoking quit attempts, success, and quit rates: a prospective study of smokers in England. *Addict Behav*. in press.

44. Selya AS, Rose JS, Dierker L, Hedeker D, Mermelstein RJ. Evaluating the mutual pathways among electronic cigarette use, conventional smoking and nicotine dependence. *Addict Abingdon Engl*. 2018;113(2):325-333. doi:10.1111/add.14013

45. Villanti AC, Feirman SP, Niaura RS, et al. How do we determine the impact of e-cigarettes on cigarette smoking cessation or reduction? Review and recommendations for answering the research question with scientific rigor. *Addiction*. 2018;113(3):391-404. doi:10.1111/add.14020

46. Etter J-F. Electronic cigarettes: a survey of users. *BMC Public Health*. 2010;10(1):231. doi:10.1186/1471-2458-10-231

47. Etter JF, Perneger TV. Attitudes toward nicotine replacement therapy in smokers and ex-smokers in the general public. *Clin Pharmacol Ther*. 2001;69(3):175-183. doi:10.1067/mcp.2001.113722

48. Brown J, Hajek P, McRobbie H, et al. Cigarette craving and withdrawal symptoms during temporary abstinence and the effect of nicotine gum. *Psychopharmacology (Berl)*. 2013;229(1):209-218. doi:10.1007/s00213-013-3100-2

49. Berg CJ, An LC, Kirch M, et al. Failure to report attempts to quit smoking. *Addict Behav*. 2010;35(10):900-904. doi:10.1016/j.addbeh.2010.06.009

50. Borland R, Partos TR, Cummings KM. Systematic Biases in Cross-sectional Community Studies may Underestimate the Effectiveness of Stop-Smoking Medications. *Nicotine Tob Res*. 2012;14(12):1483-1487. doi:10.1093/ntr/nts002

51. Levy DT, Yuan Z, Luo Y, Abrams DB. The Relationship of E-Cigarette Use to Cigarette Quit Attempts and Cessation: Insights From a Large, Nationally Representative U.S. Survey. *Nicotine Tob Res*. 2018;20(8):931-939. doi:10.1093/ntr/ntx166

52. Zhuang Y-L, Cummins SE, Sun JY, Zhu S-H. Long-term e-cigarette use and smoking cessation: a longitudinal study with US population. *Tob Control*. 2016;25(Suppl 1):i90-i95. doi:10.1136/tobaccocontrol-2016-053096

For peer review only

For peer review only - http://bmjopen.bmj.com/site/about/guidelines.xhtml

For peer review only

For peer review only - http://bmjopen.bmj.com/site/about/guidelines.xhtml

Tables

Table 1. Baseline sample characteristics

	Dual users of e-cigarettes (*n*=298)	Dual users of OTC NRT (*n*=115)	*p*
**Sociodemographic characteristics**			
Age (years), % (*n*)			
16-31	20.1 (60)	10.4 (12)	0.006
32-47	29.9 (89)	20.9 (24)	-
48-63	35.9 (107)	47.0 (54)	-
≥64	14.1 (42)	21.7 (25)	-
Female, % (*n*)	46.0 (137)	51.2 (59)	0.331
Social grade C2DE, % (*n*)	52.7 (157)	58.3 (67)	0.308
**Smoking characteristics**			
Cigarettes per day, mean (SD)	13.52 (9.56)	11.82 (6.94)	0.049
Strength of urges to smoke (range 0-5), mean (SD)	2.26 (1.03)	2.13 (1.05)	0.266
Non-daily smoker, % (*n*)	9.7 (29)	10.4 (12)	0.830
High motivation to quit, % (*n*)	23.2 (69)	35.8 (41)	0.010
Attempted to quit in past year, % (*n*)	48.6 (144)	53.6 (60)	0.375

Note. Age was categorised by standard deviation bands (16 years), with ≥80 collapsed into the 64-79 group due to low numbers. SD = standard deviation; OTC NRT = over-the-counter nicotine replacement therapy.

For peer review only

For peer review only - http://bmjopen.bmj.com/site/about/guidelines.xhtml

Table 2. Associations between use of e-cigarettes compared with use of over-the-counter nicotine replacement therapy and (i) smoking reduction, (ii) quit attempts, (iii) use of cessation aids, and (iv) use of behavioural support and/or prescription medication specifically over six-month follow-up

	Unadjusted			Adjusted*		
	**B (SE)**	**95% CI**	***p***	**B (SE)**	**95% CI**	***p***
Change in cigarettes per day	-2.15 (0.81)	-3.74; -0.56	0.008	-2.01 (0.82)	-3.62; -0.39	0.015
	**RR**	**95% CI**	***p***	**RR**	**95% CI**	***p***
Quit attempts	0.75	0.61; 0.93	0.008	0.82	0.67; 1.00	0.049
Use of evidence-based cessation aids[1]	1.04	0.92; 1.19	0.524	1.06	0.93; 1.21	0.388
Use of behavioural support and/or prescription medication[2]	0.26	0.15; 0.45	<0.001	0.27	0.16; 0.47	<0.001

Note. *All models adjusted for age, sex and social grade. The model for change in cigarettes per day also adjusted for strength of urges to smoke; the model for quit attempts also adjusted for motivation to stop smoking and past-year quit attempts.

[1] Use of e-cigarettes, over-the-counter nicotine replacement therapy, behavioural support and/or prescription medication in the most recent quit attempt reported at follow-up.

[2] Use of behavioural support and/or prescription medication in the most recent quit attempt reported at follow-up.

SE = standard error, CI = confidence interval, RR = risk ratio.

For peer review only

Supporting information captions

Table S1. Comparison of respondents lost to follow-up and analytic sample

For peer review only

For peer review only - http://bmjopen.bmj.com/site/about/guidelines.xhtml

Table S1. Comparison of respondents lost to follow-up and analytic sample

	Baseline sample (*n*=2,318)	Lost to follow-up (*n*=1,905)	Study sample (*n*=413)	*p**
**Sociodemographic characteristics**				
Age (years), % (*n*)				
16-31	31.0 (715)	33.9 (643)	17.4 (72)	<0.001
32-47	28.8 (665)	29.1 (552)	27.4 (113)	-
48-63	28.0 (646)	25.6 (485)	39.0 (161)	-
≥64	12.2 (281)	11.3 (214)	16.2 (67)	-
Female, % (*n*)	48.2 (1117)	48.3 (921)	47.5 (196)	0.743
Social grade C2DE, % (*n*)	58.9 (1366)	59.9 (1142)	54.2 (224)	0.032
**Smoking characteristics**				
Cigarettes per day, mean (SD)	11.61 (8.26)	11.29 (8.07)	13.06 (8.94)	<0.001
Strength of urges to smoke (range 0-5), mean (SD)	2.16 (1.08)	2.14 (1.09)	2.22 (1.03)	0.158
Non-daily smoker, % (*n*)	10.7 (248)	10.9 (207)	9.9 (41)	0.576
High motivation to quit, % (*n*)	27.6 (639)	27.8 (529)	26.6 (110)	0.627
Attempted to quit in past year, % (*n*)	50.9 (1159)	51.1 (955)	50.0 (204)	0.681
Product used, % (*n*)				
E-cigarettes	71.0 (1646)	70.8 (1348)	72.2 (298)	0.571
OTC NRT	29.0 (672)	29.2 (557)	27.8 (115)	-

Note. *comparison of respondents lost to follow-up and analytic sample, using t-test for means and chi-square for percentages. Age was categorised by standard deviation bands (16 years), with ≥80 collapsed into the 64-79 group due to low numbers (*n*=18 in baseline sample). SD = standard deviation; OTC NRT = over-the-counter nicotine replacement therapy.

For peer review only

STROBE Statement—Checklist of items that should be included in reports of ***cross-sectional studies***

	Item No	Recommendation	Page
**Title and abstract**	1	(*a*) Indicate the study's design with a commonly used term in the title or the abstract	1
		(*b*) Provide in the abstract an informative and balanced summary of what was done and what was found	2
**Introduction**			
Background/rationale	2	Explain the scientific background and rationale for the investigation being reported	3
Objectives	3	State specific objectives, including any prespecified hypotheses	4
**Methods**			
Study design	4	Present key elements of study design early in the paper	5
Setting	5	Describe the setting, locations, and relevant dates, including periods of recruitment, exposure, follow-up, and data collection	5
Participants	6	(*a*) Give the eligibility criteria, and the sources and methods of selection of participants	5
Variables	7	Clearly define all outcomes, exposures, predictors, potential confounders, and effect modifiers. Give diagnostic criteria, if applicable	6
Data sources/ measurement	8*	For each variable of interest, give sources of data and details of methods of assessment (measurement). Describe comparability of assessment methods if there is more than one group	6
Bias	9	Describe any efforts to address potential sources of bias	7
Study size	10	Explain how the study size was arrived at	8
Quantitative variables	11	Explain how quantitative variables were handled in the analyses. If applicable, describe which groupings were chosen and why	6
Statistical methods	12	(*a*) Describe all statistical methods, including those used to control for confounding	8
		(*b*) Describe any methods used to examine subgroups and interactions	8
		(*c*) Explain how missing data were addressed	8
		(*d*) If applicable, describe analytical methods taking account of sampling strategy	na
		(*e*) Describe any sensitivity analyses	8
**Results**			
Participants	13*	(a) Report numbers of individuals at each stage of study—eg numbers potentially eligible, examined for eligibility, confirmed eligible, included in the study, completing follow-up, and analysed	8
		(b) Give reasons for non-participation at each stage	8
		(c) Consider use of a flow diagram	na
Descriptive data	14*	(a) Give characteristics of study participants (eg demographic, clinical, social) and information on exposures and potential confounders	9
		(b) Indicate number of participants with missing data for each variable of interest	na
Outcome data	15*	Report numbers of outcome events or summary measures	Tables
Main results	16	(*a*) Give unadjusted estimates and, if applicable, confounder-adjusted estimates and their precision (eg, 95% confidence interval). Make clear which confounders were adjusted for and why they were included	Tables

For peer review only

		(*b*) Report category boundaries when continuous variables were categorized	na
		(*c*) If relevant, consider translating estimates of relative risk into absolute risk for a meaningful time period	na
Other analyses	17	Report other analyses done—eg analyses of subgroups and interactions, and sensitivity analyses	9
**Discussion**			
Key results	18	Summarise key results with reference to study objectives	10
Limitations	19	Discuss limitations of the study, taking into account sources of potential bias or imprecision. Discuss both direction and magnitude of any potential bias	12
Interpretation	20	Give a cautious overall interpretation of results considering objectives, limitations, multiplicity of analyses, results from similar studies, and other relevant evidence	12
Generalisability	21	Discuss the generalisability (external validity) of the study results	12
**Other information**			
Funding	22	Give the source of funding and the role of the funders for the present study and, if applicable, for the original study on which the present article is based	13

*Give information separately for exposed and unexposed groups.

Note: An Explanation and Elaboration article discusses each checklist item and gives methodological background and published examples of transparent reporting. The STROBE checklist is best used in conjunction with this article (freely available on the Web sites of PLoS Medicine at http://www.plosmedicine.org/, Annals of Internal Medicine at http://www.annals.org/, and Epidemiology at http://www.epidem.com/). Information on the STROBE Initiative is available at www.strobe-statement.org.

For peer review only
